# Machine learning for enzyme catalytic activity: current progress and future horizons

**DOI:** 10.1093/bib/bbag002

**Published:** 2026-01-25

**Authors:** Sizhe Qiu, Haris Saeed, Will Leonard, Feiran Li, Aidong Yang

**Affiliations:** Department of Engineering Science, University of Oxford, Parks Road, OX1 3PJ, Oxford, United Kingdom; Department of Engineering Science, University of Oxford, Parks Road, OX1 3PJ, Oxford, United Kingdom; Department of Engineering Science, University of Oxford, Parks Road, OX1 3PJ, Oxford, United Kingdom; Institute of Biopharmaceutical and Health Engineering, Tsinghua Shenzhen International Graduate School, Tsinghua University, University Town, Nanshan, 518055, Shenzhen, China; Department of Engineering Science, University of Oxford, Parks Road, OX1 3PJ, Oxford, United Kingdom

**Keywords:** deep learning, compound-protein interaction, enzyme substrate specificity, enzyme turnover number, enzyme catalytic optimum

## Abstract

Enzyme catalysis, with its advantages in environmental sustainability and efficiency, is gaining traction across diverse industrial applications, such as waste utilization and pharmaceutical biomanufacturing. However, optimizing enzyme catalytic activity remains a significant challenge. To facilitate enzyme mining and engineering, machine learning (ML) models have emerged to predict enzyme substrate specificity, enzyme turnover number, and enzyme catalytic optimum. This review endeavored to assist researchers in effectively utilizing predictive models for enzyme catalytic activity through presenting recent advancements and analyzing different approaches. We also pointed out existing limitations (e.g. dataset imbalance) and offered suggestions on potential enhancements to address them. We identified that the attention mechanism, inclusion of new features such as product information and temperature, and using transfer learning to leverage different datasets were three main useful modeling strategies. Furthermore, we envisaged that accurate predictors of enzyme catalytic activity would potentially transform enzyme and metabolic engineering, and the optimization of biocatalysis.

## Introduction

Enzyme catalysis is receiving increasing attention in chemical processes, such as carbon dioxide reduction [1], eco-friendly bio-manufacturing of chemical products [2] and agro-industrial waste utilization [3]. Compared to traditional chemical catalysis, enzyme catalysis offers key advantages including lower energy consumption, avoidance of undesirable side-reactions due to high selectivity, higher efficiency with shortened reaction routes, and long-term environmental sustainability [4–6]. Despite the comparative benefits of enzyme catalysis, the optimization of enzyme catalytic activity is still a challenging task, as natural beneficial mutations are rare and enzyme assays to screen candidate enzymes are costly [7, 8]. To overcome such obstacles, researchers have turned to computational methods to improve enzyme catalysis.

In the age of AI+biology [9], machine learning (ML)-based tools have been developed, as a promising area in computational and synthetic biology [7], to advance enzyme mining and engineering, spanning from function annotation (e.g. CLEAN [10] for enzyme class prediction) to property prediction (e.g. DeepTM [11] for enzyme thermostability). Meanwhile, some literature reviews relevant to this topic have emerged: Jiang *et al.*, 2023 [7] discussed the progress and limitations of ML models that could identify function-enhancing enzymes, including predictions of activation free energy, selectivity, kinetic parameters, etc.; Yang *et al.* 2024 [12] overviewed ML models for enzyme functional annotation and navigating the enzyme fitness landscape; Markus *et al.*, 2023 [13] focused on the application of ML on enzyme catalysis in the pharmaceutical industry; Salas-Nuñez *et al.*, 2024 [14] presented and analyzed ML-based classifiers of enzyme–substrate interactions. Nevertheless, existing works have not provided a comprehensive review on ML models that assist researchers of enzyme catalysis to first identify enzymes with the ability to catalyze target reactions, then select candidates with maximum reaction rates, and finally evaluate the effect of environmental conditions on catalytic performance.

**Figure 1:**
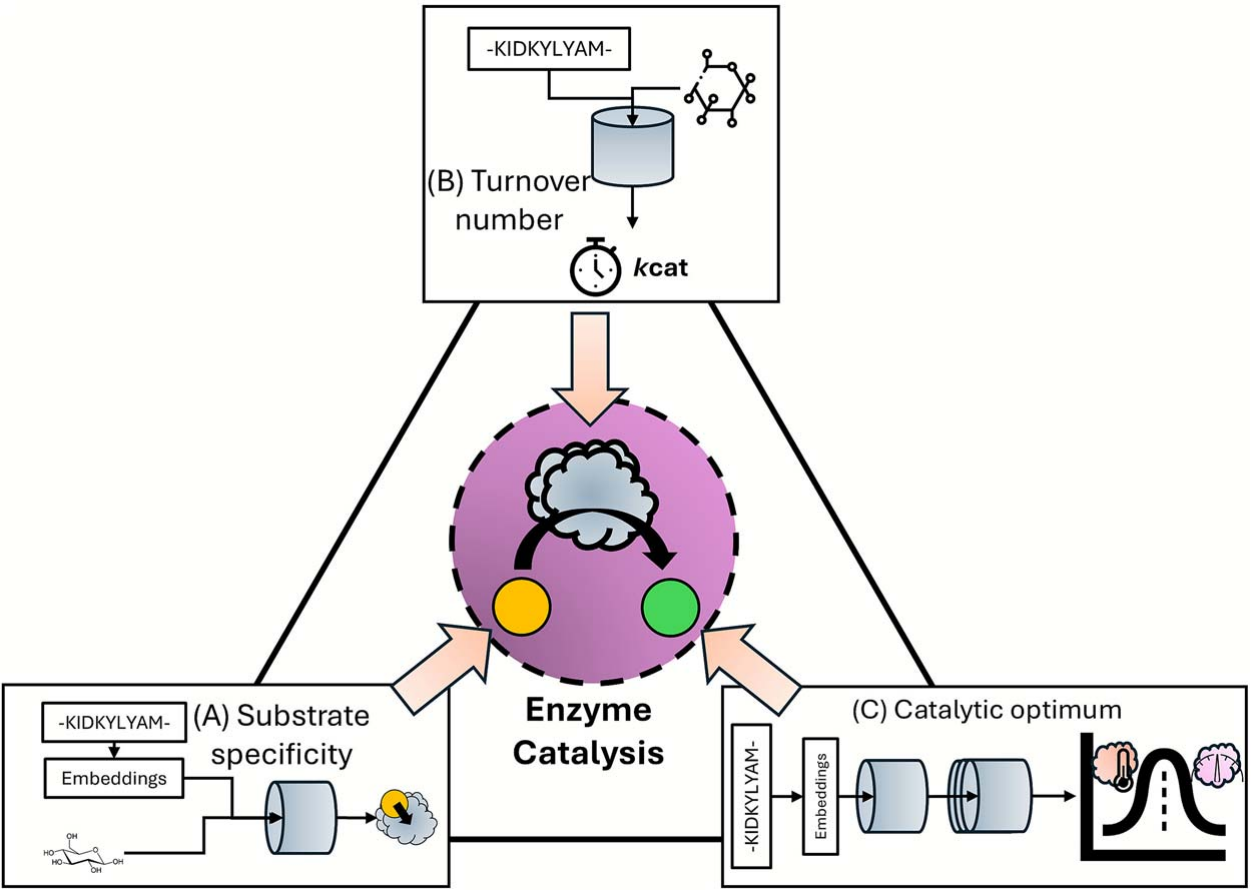
Machine learning models of three key aspects of enzyme catalytic activity. (A) Enzyme substrate specificity. (B) Enzyme catalytic rate quantified by the turnover number (${k}_{cat}$). (C) Enzyme catalytic optimum (optimal pH and temperature, $p{H}_{opt}$ and ${T}_{opt}$).

To provide a systematic review specifically for ML models of enzyme catalytic activity, this work aimed to critically evaluate models published in recent years (released before January 2025) focusing on three key questions (Fig. 1): (i) Can the enzyme catalyze the target reaction (substrate specificity)? (ii) How fast can the enzyme catalyze the target reaction (turnover number)? and (iii) Under what environmental conditions does the enzyme achieve its fastest catalytic rate (catalytic optimum)? Through analyzing different approaches and limitations of those ML models, this article intended to summarize the trends in this field and provide insights into potential directions for future advancements.

## Compound-protein interaction models of enzyme substrate specificity

Enzymes are known to have high specificities that they usually only catalyze certain types of reactions, e.g. hexokinase (EC 2.7.1.1) only catalyzes phosphorylation of six-carbon sugars. Even enzymes with promiscuity tend to have preferred substrates [15]. Traditional computational methods for predicting enzyme substrate specificity are primarily molecular docking and molecular dynamics simulations [16–18]. However, the main bottleneck of these 3D structure-based biophysical models is their high computational cost [19]. In order to efficiently determine the most suitable enzyme to catalyze a reaction, ML-based classification models have been constructed to predict the enzyme substrate specificity. For comprehensibility, this section categorized those predictive models as enzyme family-specific and general enzyme predictors.

### Enzyme family-specific predictors of enzyme substrate specificity

Enzyme family-specific predictors were mostly based on traditional ML methods, such as random forest (RF) or support vector machine (SVM), and small datasets, in contrast to general enzyme predictors. The representative models are GT_predict [20], AdenylPred [21], and Mou *et al.*, 2021 [22]. GT_predict [20] used a decision tree (DT) model to predict the sugar donor-acceptor specificity for enzymes in glycosyltransferase superfamily 1. The DT model was trained with physicochemical properties and structural information of substrates and enzyme activity screening. The cross-validation showed that GT_predict could achieve an accuracy of 90%. AdenylPred [21] used RF, naive bayes, and feedforward neural network (FNN) to predict the substrate specificity of adenylate-forming enzymes with physicochemical properties of protein sequences. The average area under the receiver operating characteristic curve (AUROC) score of AdenylPred was 0.98. The ROC curve plots the true positive rate against the false positive rate across various threshold settings [23]. Mou *et al.*, 2021 [22] used enzyme-ligand features (e.g. thermodynamic indices of the folded and extended protein state computed by [24]) to train RF, DT, logistic regression (LR), and SVM to identify the range of substrates accepted by a nitrilase. The average AUROC of Mou *et al.*, 2021 was 0.9. In contrast to GT_predict and AdenylPred discussed above, Protease-GCNN [25] was a deep learning-based classifier of substrate specificity using graph convolutional network (GCN), and the input data were residue interaction networks encoded from protease-substrate complex structures. GCN is a graph neural network (GNN) model with convolutional layers. For technical details of GNN and GCN, this review recommends *The Graph Neural Network Model* by Scarselli *et al.,* 2009 [26]. In model comparison with RF, SVM, DT, and LR, Protease-GCNN outperformed those methods with a classification accuracy above 90%. Although family-specific predictors could achieve good classification accuracy, their limited application scope made them unable to satisfy the need of high-throughput enzyme selection on massive sequencing data which contains coding sequences of different enzyme families.

### General enzyme predictors of enzyme substrate specificity

Recently, deep learning compound-protein interaction (CPI) models have been built to predict enzyme substrate specificity for general enzymes (Table 1). The inputs to most CPI models of enzyme substrate specificity discussed in this section are simplified molecular-input line-entry system (SMILES) strings of molecules and protein sequences. Enz-Pred [27] used ProSE [28], a pretrained protein language model, to transform protein sequences into embeddings, and compared different embedding methods (variational autoencoder (VAE) [29], morgan fingerprints [30], one-hot encoding [31]) of compound SMILES strings. The embedding of a protein sequence or compound SMILES string converts a string into a fixed-size vector of numbers, which enables ML algorithms to understand [32]. With extracted features from enzymes and substrates, Enz-Pred used k-nearest neighbor (KNN), FNN, and Ridge regression to perform two classification tasks: (i) the enzyme specific to a certain substrate and (ii) the substrate specific to a certain enzyme. The FNN was trained using the Adam optimizer [33] for 100 epochs. With the optimal combinations of feature extraction methods and classification algorithms, Enz-Pred could reach AUROC = 0.587 ~ 0.896 for enzyme specificity, and AUROC = 0.715 ~ 0.841 for substrate specificity. In comparison with family-specific predictors such as AdenylPred, the accuracy of Enz-Pred was relatively low. Similar to Enz-Pred, SEP-DNN [34] used ProtVec [35] and SMILESVec [36] to extract features from protein sequences and SMILES strings, respectively, and deep neural network (DNN) to classify the enzyme substrate specificity. Unfortunately, the code and data of SEP-DNN have not been made publicly available yet. Unlike Enz-Pred and SEP-DNN that used pretrained protein language models, EnzRank [37] used a trainable k-mer dictionary-based embedding method to represent protein sequence features. The encoded sequence features and morgan fingerprints of substrates were input to convolutional neural network (CNN) to classify the enzyme-substrate specificity. EnzRank had an accuracy of 80.72% on positive cases and 73.08% on negative cases.

**Table 1:** Summary of general enzyme substrate specificity classifiers

Model	Input	ML methods	Accuracy	Dataset	Link
Enz-Pred [27]	Protein sequences and compound SMILES strings	Pretrained protein language model (ProSE), VAE, morgan fingerprints, one-hot encoding, KNN, FNN, Ridge regression	Enzyme-specificity: AUROC = 0.587 ~ 0.896, Substrate-specificity: AUROC = 0.715 ~ 0.841.	60 769 entries of 6 enzyme families, train/test sets were randomly split in 10-fold cross-validation	https://github.com/samgoldman97/enz-pred
SEP-DNN [34]		Pretrained language models (ProtVec and SMILESVec), DNN	Macro F1 score = 0.966.	Positive entries of EC1–6 from KEGG [58], negative entries generated by random sampling, train/test/validation sets were randomly split with an 80–10-10 ratio	Unavailable
EnzRank [37]		Dictionary-based sequence embedding, morgan fingerprints, CNN	Classification accuracy = 80.72% (Positive), 73.08% (Negative)	11 080 positive entries from BRENDA [59], negative entries generated by random sampling, train/test/validation sets were randomly split with an 80–10-10 ratio	https://github.com/maranasgroup/EnzRank
ESP [39]		Pretrained protein language model (ESM-1b), ECFP, GNN, FCNN, gradient boosting model	AUROC = 0.956, Classification accuracy = 91.5%.	18351 positive entries from UniProt [60], negative entries generated by random sampling of substrates, train/test sets were randomly split with an 80–20 ratio	https://github.com/AlexanderKroll/ESP
ProSmith [40]		Pretrained language models (ESM-1b and ChemBERTa2), FCNN, gradient boosting model	AUROC = 0.972, Classification accuracy = 94.2%.		https://github.com/AlexanderKroll/ProSmith
FusionESP [42]		Pretrained language models (ESM-2 and MoLFormer), contrastive learning	AUROC = 0.965, Classification accuracy = 94.77%		https://github.com/dzjxzyd/FusionESP
PU-EPP [49]		GNN, bag-of-words, multi-head self-attention	AUROC = 0.985	Positive entries collected from Rhea [61], KEGG, MetaCyc [62], BRENDA, and RxnFinder [63], negative entries generated using PU learning strategy, train/test sets were randomly split in 5-fold cross-validation	https://github.com/xinghd142857/PU-EPP/
MEI [46]		Pretrained protein language model (ESM-1b), GNN, ECFP, cross-attention, FCNN	AUROC = 0.987, Classification accuracy = 96.5%	66 724 positive entries from Rhea and UniProt, negative entries generated by a transformer model, train/test sets were randomly split with a 90–10 ratio	https://github.com/KeeliaQWJ/MEI
EnzyPick [51]		Word2Vec, RXNFP, transformer with multi-head self-attention	AUROC = 0.993.	Positive and negative entries from labeled and unlabeled data in Rhea, KEGG, MetaCyc, BRENDA, and RxnFinder, train/test sets were randomly split with an 80–20 ratio	https://doi.org/10.5281/zenodo.8210150
Reactzyme [54]	Protein sequences, protein 3D structures, and compound SMILES strings	MAT-2D/3D, UniMol-2D/3D, Cross-attention, pretrained protein language model (ESM-2 and SaProt), GNN, MLP	AUROC = 0.88, Classification accuracy = 98.79%	178463 positive entries from Rhea and UniProt, negative entries generated by random sampling from enzymes and reactions of high similarity, 93–7, 95–5, and 91–9 ratios for train/test set split based on time, enzyme similarity, and reaction similarity	https://github.com/WillHua127/ReactZyme

The use of ESM [38], a state-of-the-art protein language model trained with millions of protein sequences, has been shown to be capable of effectively improving the classification accuracy of enzyme-substrate specificity. ESP [39] used ESM-1b to embed protein sequences, and GNN encoding to represent substrates. The concatenated features of protein sequences and substrates were then input to a fully connected neural network (FCNN), and the gradient boosting model was used to classify enzyme-substrate specificity. After hyperparameter optimization, ESP reached an AUROC of 0.956. ProSmith [40] also used ESM-1b to embed protein sequences, but used a pretrained model of molecules named ChemBERTa2 [41], instead of GNN, to extract features from substrates. Enzyme and substrate features were fed to a transformer network to generate classification tokens, and then, the gradient boosting model used those tokens to classify enzyme-substrate specificity. In model performance comparison on the same test set, ProSmith outcompeted ESP with a AUROC of 0.972. Subsequently, FusionESP [42] used embeddings generated by ESM-2 [43] for enzymes and MoLFormer [44] for substrates to train a contrastive learning model [45] in place of FCNN, and reached a higher classification accuracy than ProSmith on the same test set, 94.77% versus 94.2%. The advantage of contrastive learning in FusionESP was demonstrated by the model comparison with simple feature concatenation [42]. Also encoding protein sequences with ESM, MEI [46] used a pretrained CLEAN model [10] rooted in ESM-1b to generate deep representations of protein sequences. The substrate features were represented by extended-connectivity fingerprints (ECFPs) [47] and GNN encoding. Cross-attention was used to capture the interaction activity between enzymes and substrates. To understand attention mechanisms in deep learning, this review recommends *Attention Is All You Need* by Vaswani *et al.,* 2017 [48]. The deep representations and interaction features of enzymes and substrates were fed to the FCNN to classify enzyme substrate specificity. MEI achieved a classification accuracy of 96.5%.

One bottleneck of predicting enzyme-substrate specificity lies in the lack of negative samples, where the enzyme catalysis cannot happen. To tackle this issue, PU-EPP [49] conducted positive unlabeled learning iteratively during the training process to make use of massive unlabeled samples by removing potential positive samples. The enzyme sequences were encoded by the bag-of-words model [50], and the molecular graphs of substrates were encoded by GNN. Multi-head self-attention was employed to represent enzyme-substrate interactions. PU-EPP reached an AUROC of 0.985.

None of the CPI models of enzyme substrate specificity discussed above included the information of products or representations of reactions converting substrates to products. EnzyPick [51] resolved this limitation by using reaction fingerprints from complete chemical reactions (substrate–product pairs) computed by RXNFP [52] as an input feature. RXNFP is a transformer-based model trained to classify chemical reaction classes from SMILES string representations of reactions (e.g. ‘[substrate A].[substrate B]>>[product C].[product D]’). The numerical representations of substrate–product pairs were concatenated with protein sequence features generated by Word2Vec [53]. Then, the substrate–product–enzyme features were input to a modified transformer architecture with multi-head self-attention mechanism, and the AUROC score achieved by EnzyPick was 0.993. Reactzyme [54] was another classification model of enzyme–substrate–product specificity. It encoded molecular features of substrates and products with MAT-2D/3D [55] and UniMol-2D/3D [56], and then computed cross attention between substrates and products to represent the transition in the reaction. Enzyme sequences were encoded by ESM-2, SaProt [57], and GNN. Subsequently, encoded enzyme and reaction features were input to MLP, instead of complicated models like XGBoost, to classify the enzyme substrate specificity. Reactzyme had a classification accuracy of 98.79%.

**Figure 2:**
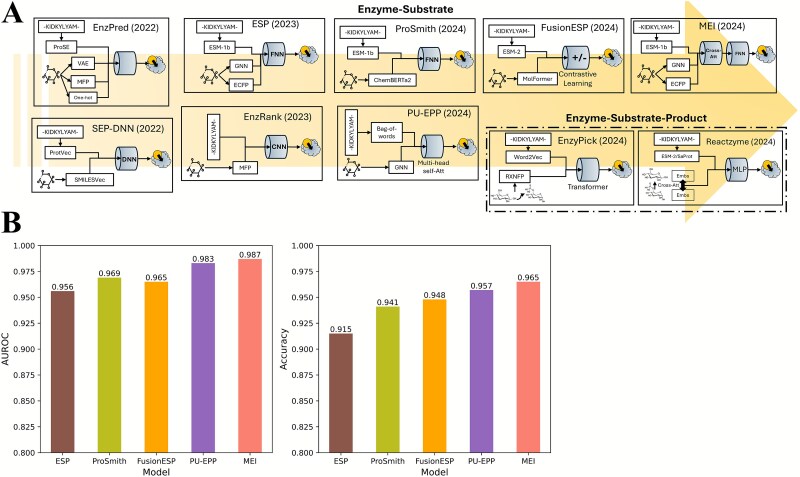
(A) Enzyme substrate specificity classifiers developed in recent years. VAE: Variational autoencoder, MFP: Molecular fingerprint, ECFP: Extended-connectivity fingerprint, GNN: Graph neural network, DNN: Dense neural network, FNN: Fully connected neural network, Att: Attention mechanism. (B) Prediction accuracy comparison of representative enzyme substrate specificity classifiers on the train and test datasets of ESP. AUROC: Area under the receiver operating characteristic curve.

For representative CPI models of enzyme substrate specificity that are based on protein sequences and compound SMILES strings, this review provided a benchmark analysis using the training and test datasets of ESP [39] to evaluate their prediction performances under default hyperparameter settings (Fig. 2B). The benchmark results, largely consistent with previously reported accuracies (Table 1), showed that these models (e.g. ProSmith) can achieve classification accuracies above 90%, although the performance is sometimes limited by dataset coverage, especially for unseen compounds (new molecules) and protein sequences with low identity to the training dataset [39]. With these predictive models, enzymes discovered from sequencing data can be annotated for their catalytic specificities.

## Compound-protein interaction models of enzyme turnover numbers

**Figure 3:**
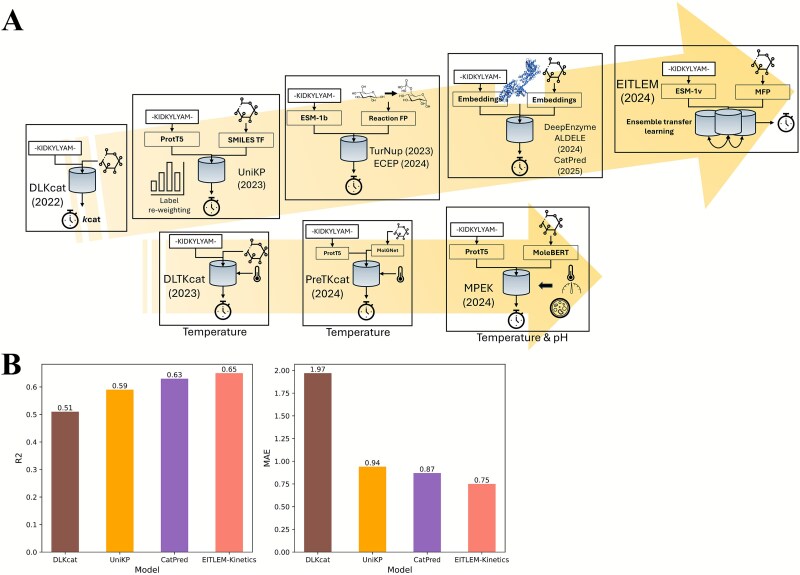
(A) Enzyme ${k}_{cat}$ prediction models developed in recent years. SMILES TF: SMILES transformer, FP: Fingerprint, MFP: Molecular fingerprint. (B) Prediction accuracy comparison of representative enzyme ${k}_{cat}$ predictors on the CatPred-DB dataset. R2: R-squared, MAE: Mean average error.

Enzyme turnover number (${k}_{cat}$) is the most commonly used quantitative measure of the speed of enzyme catalyzed reactions, and is defined as the number of maximum substrate molecules converted by the enzyme molecule per unit time (${k}_{cat}=\frac{v_{max}}{\left[{E}_T\right]}$, ${v}_{max}$: maximum reaction rate, $\left[{E}_T\right]$: total enzyme concentration) [64]. Various computational methods have been developed to estimate enzyme ${k}_{cat}$ values. The most direct approach is to compute apparent catalytic rate (${k}_{app}$) through dividing measured reaction fluxes by quantified protein abundance levels [65, 66]. The main shortcoming of computing ${k}_{app}$ is the high cost of measuring fluxomics and proteomics. Compared to direct calculation, using ML to predict enzyme ${k}_{cat}$ is an expedient solution. Heckmann *et al.*, 2018 [67] trained an ensemble regressor of ElasticNet, RF and artificial neural network (ANN) to predict ${k}_{cat}$ values of metabolic enzymes of *E*scherichia* coli* K12 strain, with enzyme biochemistry, protein structure, and metabolic network context as input features. Heckmann *et al.*, 2018 achieved an accuracy of R-squared (R2) (log10-scale) =0.31, but the required input features (e.g. metabolite concentrations, reaction fluxes computed by flux balance analysis) largely restricted the scope of this model. Similar to the prediction of enzyme–substrate specificity, researchers adopted the CPI modeling framework to enhance both the accuracy and generality of enzyme ${k}_{cat}$ prediction (Table 2, Fig.  3A).

**Table 2:** Summary of maximum enzyme ${k}_{cat}$ predictors

Model	Input	ML methods	Accuracy (log10-scale)	Dataset	Link
DLKcat [68]	Protein sequences and compound SMILES strings	GNN and attention CNN	R2 = 0.44, RMSE = 1.06	16 838 entries from BRENDA and SABIO-RK [86], train/test/validation sets were randomly split with an 80–10-10 ratio	https://github.com/SysBioChalmers/DLKcat
UniKP [70]		Pretrained language models (ProtT5-XL-UniRef50 and SMILES transformer), Extra Trees model	R2 = 0.65, RMSE = 0.89	16 838 entries from BRENDA and SABIO-RK, train/test sets were randomly split with a 90–10 ratio	https://github.com/Luo-SynBioLab/UniKP
TurNuP [75]		Pretrained protein language model (ESM-1b), reaction fingerprints (structural, differential, and difference), XGBoost	R2 = 0.44, RMSE = 0.9	4271 entries from BRENDA, UniProt, and SABIO-RK, train/test sets were randomly split with an 80–20 ratio	https://github.com/AlexanderKroll/Kcat_prediction
DeepEnzyme [78]	Protein sequences, protein 3D-structures, and compound SMILES strings	Attention GCN	R2 = 0.6, RMSE = 0.95	11 927 entries of the DLKcat dataset with entries of high similarity removed, train/test/validation sets were randomly split with an 80–10-10 ratio	https://github.com/hongzhonglu/DeepEnzyme
CatPred [81]	Protein sequences and compound SMILES strings	Pretrained protein language model (ESM-2), self-attention, and GNN	R2 = 0.608, MAE = 0.703	23197 entries from BRENDA and SABIO-RK, train/test/validation sets were randomly split with an 80–10-10 ratio	https://github.com/maranasgroup/CatPred
ECEP [76]		Pretrained protein language model (ESM-1b), reaction fingerprints (structural, differential, and difference), CNN	R2 = 0.54, RMSE = 0.68	A train set with 3391 entries, and a test set with 874 entries from BRENDA, UniProt, and SABIO-RK	https://github.com/misharisaud/ECEP
ALDELE [79]	Protein sequences, protein 3D-structures, and compound SMILES strings	ANN, GNN, CNN, attention mechanism	PCC = 0.82, RMSE = 1.02 (log-2 scale RMSE = 3.39)	16808 entries from BRENDA and SABIO-RK, train/test sets were randomly split in 5-fold cross-validation	https://github.com/Xiangwen-Wang/ALDELE
EITLEM-Kinetics [82]	Protein sequences and compound SMILES strings	Pretrained protein language model (ESM-1v), molecular fingerprints, attention mechanism, ensemble iterative transfer learning	R2 = 0.721, RMSE = 0.825, MAE = 0.510	34429 entries from BRENDA and SABIO-RK, train/test/validation sets were randomly split with an 80–10-10 ratio	https://github.com/XvesS/EITLEM-Kinetics

### Predicting maximum enzyme turnover numbers

As the first CPI model of enzyme ${k}_{cat}$, DLKcat [68] successfully reduced the prediction error to around one order of magnitude (log10-scale root mean squared error (RMSE) =1.06 and R2 (log10-scale) =0.44). DLKcat used the GNN to extract molecular features from substrates, and the attention CNN to extract protein residue features from 3-mers of enzyme proteins. The attention weights were computed as a learnable matrix transformation of molecular features to capture the interactions of important residues with substrates. Substrate molecular features and protein residue features were concatenated and then used to regress for the $lo{g}_{10}{k}_{cat}$ via linear layers. The attention weights on protein residues rendered interpretability to DLKcat, and the analysis of residue attention weights showed that DLKcat could capture effective mutations. Nevertheless, DLKcat was criticized for its inferior accuracy with enzymes of low similarity to those in the training dataset [69].

Subsequently, several CPI models were developed to further reduce the prediction error of ${k}_{cat}$, as summarized in Table 2, with various strategies such as sample weight redistribution and inclusion of protein 3D structures. UniKP [70] used Label Distribution Smoothing (LDS) [71] to successfully mitigate the imbalance in data and reduced the prediction error in high-value ${k}_{cat}$ prediction tasks. It used ProtT5-XL-UniRef50 [72] and SMILES transformer [73] to generate embeddings for enzyme proteins and substrates, and the concatenated features were input to the Extra Trees model [74]. UniKP outperformed DLKcat and achieved an accuracy of R2 = 0.65, RMSE = 0.89 for ${k}_{cat}$ (log10-scale). Notably, UniKP could also predict Michaelis constant (${K}_m$) and specificity constant (${k}_{cat}/{K}_m$) with high accuracy (log10-scale RMSE (${K}_m$) = 0.8, log10-scale RMSE (${k}_{cat}/{K}_m$) = 1.07) [70].

Different from classic CPI models such as DLKcat and UniKP, TurNuP [75] and ECEP [76], as predictors of reaction-level ${k}_{cat}$, further included the information of products in addition to enzymes and substrates. In both TurNuP and ECEP, structural, differential, and difference reaction fingerprints were computed using RDKit [77], and protein sequence embeddings were generated by ESM-1b. After feature concatenation, TurNuP [75] used XGBoost to regress for ${k}_{cat}$ and obtained an accuracy of R2 = 0.44, RMSE = 0.9 (log10-scale). The authors of ECEP [76] found that CNN performed better than XGBoost, and ECEP achieved an accuracy of R2 = 0.54, RMSE = 0.68 (log10-scale).

The inclusion of protein 3D structures in DeepEnzyme [78] allowed the attention graph convolutional neural network (GCN) to extract features from both substrate molecular graphs and protein residue networks. DeepEnzyme outperformed DLKcat and TurNup, and achieved an accuracy of R2 = 0.6, RMSE = 0.95 (log10-scale). ALDELE [79] also used protein 3D structures as input features, but employed a different feature representation method: CNN representation of Rosetta energy scores [80]. ALDELE achieved an accuracy of Pearson correlation coefficient (PCC) = 0.82 and log-2 scale RMSE = 3.39. Nevertheless, the authors of CatPred [81] found that GNN encoded protein 3D structures could not provide any improvements on top of multi-head self-attention and pretrained protein language model (ESM-2). The substrate molecules were encoded by GNN in CatPred, and concatenated with protein sequence features as the input to a probabilistic regression model. In model performance comparison, CatPred outperformed DLKcat and UniKP on its hold-out test set with R2 = 0.608, mean average error (MAE) = 0.703 (log10-scale). Therefore, it remains uncertain whether incorporating protein structures can enhance the prediction performance of ${k}_{cat}$.

The most recent advancement of ${k}_{cat}$ prediction was the use of ensemble iterative transfer learning in EITLEM-Kinetics [82]. EITLEM-Kinetics consisted of three predictors, ${k}_{cat}$, ${K}_m$, and ${k}_{cat}/{K}_m$. The predictor of ${k}_{cat}/{K}_m$ is an ensemble model of ${k}_{cat}$ and ${K}_m$ models. Protein sequence embeddings were computed by ESM-1v, and molecular fingerprints were computed by MACCSKeys [83]. Attention weighted features of enzymes and substrates were computed to represent interactions between atoms and residues, and then passed to a multi-head attention aggregation module to generate a concatenated feature vector. Then, FNN was used to regress for the target value. To boost the prediction accuracy, the ensemble iterative transfer learning strategy was employed to finetune model parameters for ${k}_{cat}$, ${K}_m$, and ${k}_{cat}/{K}_m$. In each iteration, parameters of ${k}_{cat}$ and ${K}_m$ models were extracted to train the ensemble model of ${k}_{cat}/{K}_m$, and then, the trained parameters of ${k}_{cat}$ and ${K}_m$ from the ensemble model were extracted to retrain ${k}_{cat}$ and ${K}_m$ models. After 7 iterations, the R2 scores of ${k}_{cat},{K}_m, and\ {k}_{cat}/{K}_m$ increased by 10% ~ 30%.

With the improvement of enzyme ${k}_{cat}$ prediction accuracy, deep learning-based ${k}_{cat}$ predictors have been increasingly applied to enhance the enzyme catalytic rate through *in-silico* enzyme screening and the rational design of mutations. As traditional enzyme mining and evolution are costly in time and labour, Yu *et al.*, 2023 used UniKP to select 5 tyrosine ammonia lyases (TALs) with the highest predicted ${k}_{cat}$ values from 1000 homologs, and AsTAL from *Armillaria solidipes* showed the highest ${k}_{cat}$ value [70]. In addition, UniKP was used to predict ${k}_{cat}$ values for all possible single-point mutants of the TAL from *Rhodotorula glutinis*, and successfully identified two effective mutations. Similarly, Liu *et al.*, 2023 selected effective mutations of β-Ketothiolase based on enzyme ${k}_{cat}$ predicted by DLKcat to reduce the workload of mutagenesis [84]. As another application case of *in-silico* enzyme screening for optimized catalytic activity, Xu *et al.*, 2025 used DLKcat and TurNup together to score around 5000 enzyme sequences homologous to *Dickeya parazeae* aldehyde dehydrogenase to find top 100 candidates [85]. Among these candidates, acetaldehyde dehydrogenase from *Buttiauxella sp. S04-F03* exhibited 14.1-fold higher catalytic activity than *Dickeya parazeae* aldehyde dehydrogenase. In brief, these application cases demonstrated the practical value of deep learning-based ${k}_{cat}$ predictors.

### Incorporating temperature and pH into enzyme turnover number prediction

Although most CPI models of enzyme ${k}_{cat}$ discussed above could achieve prediction errors within one order of magnitude (log10-scale RMSE <1), they could not account for the strong dependencies of enzyme activity on temperature and pH [87–89], thereby limiting their predictive performance. Several studies managed to incorporate temperature and pH in ${k}_{cat}$ prediction for certain enzyme families, such as hydrolases [90, 91]. EF-UniKP [70], modified from UniKP, was developed to predict temperature dependent and pH dependent ${k}_{cat}$ values separately, but the datasets used by those two models were too small (n = 636 for pH, n = 572 for temperature) to generalize the impact of pH and temperature on enzyme ${k}_{cat}$ (R2 = 0.4 for temperature dependent ${k}_{cat}$ and R2 = 0.45 for pH dependent ${k}_{cat}$). In short, those approaches could not provide accurate predictions for enzyme ${k}_{cat}$ under different environmental conditions.

Existing CPI models of condition-dependent enzyme ${k}_{cat}$ with relatively good accuracy were DLTKcat [92], PreTKcat [93], and MPEK [94], the former two for temperature-dependent enzyme ${k}_{cat}$ and the latter one for both pH and temperature-dependent enzyme ${k}_{cat}$ (Table 3, Fig. 3A). DLTKcat [92] captured bi-directional attention weights between enzyme protein residues and substrate atoms, and included temperature and inverse of temperature as features to make ${k}_{cat}$ prediction sensitive to temperature changes. DLTKcat reached a prediction accuracy of R2 = 0.66, RMSE = 0.88 (log10-scale) for temperature dependent ${k}_{cat}$, and demonstrated the significant feature importance of temperature. However, DLTKcat had a limitation that its dictionary-based encoding of protein sequences and substrates could not work for residue 3-mers, molecular fingerprints, atoms, and chemical bonds absent from its training dataset. Also, the oversampled training dataset of DLTKcat had data leakage [93]. Subsequently, PreTKcat [93] used pretrained language models of protein sequences and substrates, ProT5 [72] and MolGNet [95], to improve the prediction performance of temperature dependent ${k}_{cat}$. After the encoding by pretrained language models, the mean pooling of residue features, sum pooling of atom features, temperature, and inverse of temperature were concatenated as the input to Extra Trees Model to regress for temperature dependent ${k}_{cat}$. PreTKcat achieved an accuracy of R2 = 0.69 and RMSE = 0.85, and provided a benchmark model for enzyme kinetics.

**Table 3:** Summary of temperature and pH-dependent enzyme ${k}_{cat}$ predictors

Model	Input	ML methods	Accuracy (log10-scale)	Dataset	Link
EF-UniKP (Temperature) [70]	Protein sequences, compound SMILES strings, and temperature	Pretrained language models (ProtT5 and SMILES transformer), Extra Trees model	R2 = 0.4	572 entries from UniProt, train/test sets were randomly split with an 80–20 ratio	https://github.com/Luo-SynBioLab/UniKP
EF-UniKP (pH) [70]	Protein sequences, compound SMILES strings, and pH	Pretrained language models (ProtT5 and SMILES transformer), Extra Trees model	R2 = 0.45	636 entries from UniProt, train/test sets were randomly split with an 80–20 ratio	https://github.com/Luo-SynBioLab/UniKP
DLTKcat (Temperature) [92]	Protein sequences, compound SMILES strings, and temperature	Attention GNN, CNN, and bi-directional attention	R2 = 0.66, RMSE = 0.88	4383 entries from SABIO-RK and 11,866 entries from BRENDA, oversampling for entries at low (T < 20°C) and high (T > 40°C) temperature ranges, train/test/validation sets were randomly split with an 80–10-10 ratio	https://github.com/SizheQiu/DLTKcat
PreTKcat (Temperature) [93]		Pretrained language models (ProtT5 and MolGNet), Extra Trees model	R2 = 0.69, RMSE = 0.85	The dataset of DLTKcat was randomly split into train/test sets in 10-fold cross-validation	https://github.com/MrVincentCai/PreTKcat
MPEK (Temperature and pH) [94]	Protein sequences, compound SMILES strings, temperature, pH, and organismal information	Pretrained language models (ProtT5 and Mole-BERT) and CGC framework	R2 = 0.648, RMSE = 0.594	14237 entries containing both ${k}_{cat}$ and ${K}_m$ values from BRENDA and SABIO-RK, train/test/validation sets were randomly split with an 80–10-10 ratio	https://github.com/kotori-y/mpek

MPEK [94] was a multi-task predictor of both ${k}_{cat}$ and ${K}_m$, and its dataset included temperature, pH and organismal information. MPEK extracted protein sequence features using ProtT5 and substrate molecular features using Mole-BERT [96]. Radial basis functions [97] and one-hot-encoding were used to encode organismal information, pH, and temperature. All encoded features were concatenated to train a customized gate control (CGC) model, consisting of an expert layer and a tower layer, to predict both ${K}_m$ and ${k}_{cat}$ simultaneously. The accuracy reached by MPEK was R2 = 0.648, RMSE = 0.594 (log10-scale) for ${k}_{cat}$ and R2 = 0.606, RMSE = 0.629 (log10-scale) for ${K}_m$. Despite its superior performance, MPEK had low interpretability due to its lack of feature importance analysis functions (e.g. protein residue attention weight analysis in DLKcat [68]).

Thus far, CPI models of enzyme ${k}_{cat}$ have now reached an error within one order of magnitude. For representative enzyme ${k}_{cat}$ predictors that are based on protein sequences and compound SMILES strings, this review provided a benchmark analysis using the CatPred-DB dataset [81] (randomly split into train and test sets with a 9:1 ratio) to evaluate their prediction performances under default hyperparameter settings (Fig. 3B). The benchmark results were largely consistent with the previously reported prediction accuracies (Table 2), and demonstrated the performance improvements enabled by pretrained protein language models. In brief, the use of transfer learning affords EITLEM-Kinetics a superior accuracy on maximum ${k}_{cat}$, while MPEK is currently the most accurate predictor of temperature and pH dependent-enzyme ${k}_{cat}$. Moreover, a few of these predictors have already been successfully used in enzyme mining and engineering, such as UniKP [70] and DLKcat [84, 85].

## Protein sequence-based prediction of enzyme catalytic optimum

**Figure 4:**
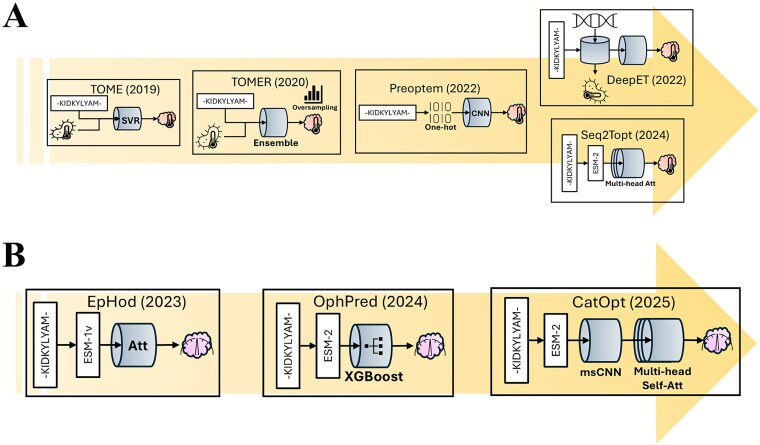
(A) The recent advancements of sequence-based ML models of enzyme optimal temperature. Att: Attention mechanism, msCNN: Multi-scale CNN. (B) the recent advancements of sequence-based ML models of enzyme optimal pH.

While enzyme ${k}_{cat}$ quantifies the reaction rate, enzyme catalytic optimum, specifically the enzyme optimal temperature (${T}_{opt}$) and pH ($p{H}_{opt}$), determines the condition where the highest catalytic rate is reached. Therefore, computational predictions of enzyme ${T}_{opt}$ and $p{H}_{opt}$ can facilitate enzyme mining and engineering for specific industrial applications [98] by allowing researchers to circumvent the extensive resource demands of traditional experimental enzyme screening. The most commonly used traditional computational method to determine enzyme ${T}_{opt}$ and $p{H}_{opt}$ is molecular dynamics simulation [99, 100], and the bottleneck, like in enzyme substrate specificity prediction (section 2), is still high computational cost [19, 101]. Therefore, ML models of enzyme ${T}_{opt}$ and $p{H}_{opt}$ have been developed to facilitate high-throughput prediction of enzyme catalytic optimum, and these models have evolved from traditional ML methods [e.g. support vector regression (SVR)] to deep learning methods (Tables 4 and 5, Fig. 4).

**Table 4:** Summary of enzyme ${T}_{opt}$ predictors

Model	Input	ML methods	Accuracy	Dataset	Link
TOME [102]	Protein sequences and OGTs	SVR	R2 = 0.94, RMSE = 4.46°C (median = 37°C)	2609 enzyme sequences and ${T}_{opt}$ from UniProt and BRENDA, train/test sets were randomly split in 5-fold cross-validation	https://github.com/EngqvistLab/Tome
TOMER [103]		Synthetic minority over-sampling, ensemble averaging	R2 = 0.632 (${T}_{opt}$>85°C)		http://github.com/jafetgado/tomer
Preoptem [104]	Protein sequences	One-hot encoding and CNN	R2 = 0.36 MAE = 9.62°C	Unavailable	https://github.com/BRITian/Preoptem
DeepET [105]		Transfer learning on OGT, and RNN	R2 = 0.57 RMSE = 12.2°C (median = 45°C)	The dataset of TOME, train/test sets were randomly split with a 90–10 ratio	https://zenodo.org/records/6351465
Seq2Topt [108]		Pretrained language model of proteins (ESM-2), multi-head attention and residual dense network	R2 = 0.57, RMSE = 12.26 °C	The dataset of TOME with oversampling for entries at ${T}_{opt}$>80°C, train/test/validation sets were randomly split with an 80–10-10 ratio	https://github.com/SizheQiu/Seq2Topt

**Table 5:** Summary of enzyme $p{H}_{opt}$ predictors

Model	Input	ML methods	Accuracy	Dataset	Link
EpHod [112]	Protein sequences	Pretrained protein language model (ESM-1v), lightweight attention, and residual dense network	R2 = 0.399, RMSE = 0.895, MAE = 0.656	9855 enzyme sequences and $p{H}_{opt}$ from UniProt and BRENDA, 20% sequences randomly selected as the test set, 10% of the remaining sequences randomly selected as the validation set	https://github.com/jafetgado/EpHod
Seq2pHopt [108]		Pretrained language model of proteins (ESM-2), multi-head attention and residual dense network	R2 = 0.369, RMSE = 0.917		https://github.com/SizheQiu/Seq2Topt
OphPred [113]		Pretrained protein language model (ESM-2) and XGBoost	R2 = 0.458 RMSE = 0.85, MAE = 0.616		https://github.com/i-Molecule/optimalPh
CatOpt [114]		Pretrained protein language model (ESM-2), multi-scale CNN, multi-head self-attention, and residual dense network	R2 = 0.479, RMSE = 0.833, MAE = 0.607		https://github.com/SizheQiu/CatOpt

### Prediction of enzyme optimal temperature

TOME [102], as the earliest predictor of ${T}_{opt}$ for general enzymes, achieved an accuracy of R2 = 0.94 and RMSE = 4.46°C when predicting enzyme ${T}_{opt}$ for a dataset with a median ${T}_{opt}$=37 °C. TOME utilized SVR to predict optimal growth temperatures (OGTs) of microorganisms as a preliminary predictor given the proteomes of host microorganisms, and then used RF to predict enzyme ${T}_{opt}$ with protein sequences and OGTs. Although TOME was effective, it had two limitations: (i) the requirement of OGTs, (ii) low accuracy in the high ${T}_{opt}$ value range. For ${T}_{opt}$ exceeding 85°C, the RMSE was higher than 10°C [103]. TOMER [103] was developed to address this second limitation. TOMER used synthetic minority over-sampling to mitigate the imbalance in the training dataset, and ensemble averaging of different ML models (SVR, KNN, ElasticNet, and Bayesian ridge regression) to regress for enzyme ${T}_{opt}$ values. TOMER raised the R2 of enzyme ${T}_{opt}$ prediction for ${T}_{opt}>{85}^{{}^{\circ}}C$ from 0.527 with TOME to 0.632, although the requirement of OGTs remained.

Subsequently, researchers of this field aimed to build models to achieve or exceed the performance of TOMER without OGTs for enzyme ${T}_{opt}$ prediction (Fig. 4A). Preoptem [104] utilized a CNN with one-hot encoding for protein sequences, the sole input. However, it could only reach R2 = 0.36 on its test dataset. As a more advanced approach, DeepET [105] used a residual neural network (RNN) consisting of CNNs and residual connection blocks to predict ${T}_{opt}$ from solely protein sequences, but employed transfer learning to address the impact of limited training data. First, one-hot encoded protein sequences were used to predict OGTs of host microorganisms, and this training task had a much larger dataset (around 3 million enzymes) than enzyme ${T}_{opt}$. Then, the sequence embeddings of the OGT predictor were used to predict enzyme ${T}_{opt}$. Transfer learning allowed DeepET to make use of the sequence information from a related dataset. Various transfer learning approaches were assessed, such as resetting weights across CNN layers and fine-tuning the pretrained model. The best performance (R2 = 0.57 and RMSE = 12.2°C) on its hold-out test set (median = 45°C) was reached when tuning the last two dense layers but freezing the convolution layers. In the comparison of different sequence encoding methods, transfer learning from OGTs outcompeted iFeatures [106] and UniRep [107].

The most recently published enzyme ${T}_{opt}$ predictor, Seq2Topt [108], took an alternative approach of transfer learning, that was sequence embedding by a pretrained language model of proteins. Seq2Topt used ESM-2 to generate protein sequence embeddings, which were input to multi-head attention for attention weighted features. Then, attention weighted features were fed to residual dense neural networks for the regression of enzyme ${T}_{opt}$. With the protein sequence as the sole input, Seq2Topt outperformed DeepET and Preoptem with RMSE = 12.26°C and R2 = 0.57 on its own hold-out test set. Furthermore, the model architecture of Seq2Topt gave rise to Seq2pHopt for enzyme $p{H}_{opt}$ prediction (RMSE = 0.88, R2 = 0.42) and Seq2Tm for enzyme melting temperature (${T}_m$) prediction (RMSE = 7.57 °C, R2 = 0.64), suggesting the broad applicability of Seq2Topt’s architecture for enzyme property prediction. The superior performance of DeepET and Seq2Topt demonstrated the potential of transfer learning in advancing predictive accuracy of enzyme catalytic optimum, as in enzyme substrate specificity and enzyme ${k}_{cat}$.

### Prediction of enzyme optimal pH

Some of the earliest ML approaches for $p{H}_{opt}$ prediction aimed at binary classification of $p{H}_{opt}$ as alkaline or acidic. For example, AcalPred [109] used an amino acid occurrence-based vector approach for residue identification and SVM to classify alkaline and acidic enzymes. AcalPred achieved an impressive F1 score of 0.97. Another early method, Zhang *et al.*, 2009 [110], used protein secondary structure predicted by PREDATOR [111] as the input feature to classify alkaline and acidic enzymes with RF, and achieved an F1 score of 0.907.

Recent approaches have employed deep learning techniques, leveraging protein language models, to enhance the prediction accuracy of enzyme $p{H}_{opt}$ values (Table 5**,**  Fig. 4B). EpHod [112] utilized a semi-supervised learning approach, combining ESM-1v encoding of protein sequences with a lightweight attention mechanism and residual dense neural network, achieving an accuracy of R2 = 0.399 and RMSE = 0.895. A key aspect of the success of EpHod was the utilization of a related dataset, the ESM-1v model was pretrained on a separate dataset containing 1.7 million proteins with environmental pH values (i.e. for host microorganisms). The model’s ability to learn biophysical features related to enzyme $p{H}_{opt}$, such as the proximity of residues to the catalytic centre and solvent accessibility, further highlighted the power of transfer learning [112].

Building upon the success of EpHod, OphPred [113] was developed using ESM-2 embedding of protein sequences and XGBoost for regression. OphPred achieved an R2 of 0.458 and an RMSE of 0.85 on the test set of EpHod. The model’s performance was noteworthy as it used only protein sequences as the input, without requiring additional structural or functional data. OphPred’s use of ESM-2 encoding and XGBoost regression showcased the effectiveness of combining state-of-the-art protein language models with traditional ML algorithms.

The most recent advancement in $p{H}_{opt}$ prediction was CatOpt [114]. CatOpt consisted of ESM-2 embedding of protein sequences, multi-head self-attention, and residual dense neural networks. CatOpt outperformed EpHod and OphPred on the same test and train sets, with an accuracy of RMSE = 0.833 and R2 = 0.479. Apart from the structural and evolutionary knowledge transferred by ESM-2 encoding, multi-head self-attention in CatOpt modeled the dependencies of different protein sequence regions, and could provide interpretability on protein residues. Furthermore, CatOpt was applied to screen all possible single-point mutations close to substrate binding sites of Pyrococcus horikoshii diacetylchitobiose deacetylase (PhDac) for the lowest enzyme $p{H}_{opt}$, and identified two mutation sites that enhanced the catalytic activity of PhDac at low pH values [114]. The rational design of diacetylchitobiose deacetylase by CatOpt demonstrated its potential in aiding enzyme engineering.

To sum up, sequence-based predictive models of enzyme ${T}_{opt}$ and $p{H}_{opt}$ have achieved RMSE = ~10°C and RMSE = ~0.8, respectively, although there still exist limitations like dataset imbalance. For benchmark analyses of all enzyme ${T}_{opt}$ and $p{H}_{opt}$ predictors, please refer to the model comparison sections in the Seq2Topt [108] and CatOpt [114] papers, which reported results consistent with those in Tables 4 and 5. Advancements in prediction accuracy of enzyme ${T}_{opt}$ and $p{H}_{opt}$ have demonstrated the viability of ML model-based selection of candidate enzymes for various working conditions, and ML model-guided enzyme engineering to alter the catalytic optimum (e.g. CatOpt [114]).

## Key insights and outlook

This review highlighted recent advancements in ML models on enzyme catalytic activity in three key areas, i.e. CPI models of enzyme–substrate specificity, CPI models of enzyme turnover numbers, and sequence-based models of enzyme catalytic optimum. Currently, the binary classification of enzyme-substrate specificity has reached an AUROC above 0.95, the prediction error of enzyme ${k}_{cat}$ has been reduced to within one order of magnitude (log10-scale RMSE<1), and enzyme ${T}_{opt}$ and $p{H}_{opt}$ can be accurately predicted with average errors close to 10°C and 1, respectively. Furthermore, several ML models have been used in practices to assist enzyme mining and engineering, such as DLKcat [68], UniKP [70], TOME [115], and CatOpt [114]. Collectively, the recent advancements in these state-of-the-art models underscore the potential of ML in predicting and optimizing enzyme catalytic activity, thereby paving the way for accelerated progress in applications of enzyme catalysis in both academia and industry.

Recent enhancement in prediction accuracy across three types of ML models in recent years can be attributed to three main strategies: (i) attention mechanism, (ii) additional feature inclusion (e.g. product information), (iii) transfer learning. In CPI models, the attention mechanism was used to represent interactions between residues of enzyme proteins and atoms of substrate molecules through learnable weights, emblematically demonstrated by DLKcat, DLTKcat, and EITLEM-Kinetics. Meanwhile, lightweight attention and self-attention were used in sequence-based models of enzyme ${T}_{opt}$ and $p{H}_{opt}$ [112, 114]. As a representative example, CatOpt used multi-head self-attention to model the dependencies of different protein sequence regions, and outperformed EpHod and OphPred [114]. Inclusion of new features can also improve the prediction performance. For example, EnzyPick used reaction fingerprints to extract features from both substrates and products; MPEK included temperature, pH, and organismal information as additional features, and outperformed previously published models of enzyme ${k}_{cat}$. The most remarkable method is transfer learning with pretrained models of protein sequences and molecules. As the prediction accuracy is often restricted by the dataset size [39, 69], transfer learning can provide knowledge from other relevant datasets to enhance the performance. For instance, ESM was used to transfer structural and evolutionary information from much larger datasets in all three types of ML models on enzyme catalytic activity. One of the most noteworthy applications of transfer learning is the ensemble iterative transfer learning in EITLEM-Kinetics (**section 3.1**). In short, those three methods have been demonstrated to be effective in ML models presented in this review.

Despite the significant progress made in ML-based modeling of enzyme catalytic activity, several challenges remain, limiting further improvements in prediction accuracy. Predicting temperature and pH dependent enzyme ${k}_{cat}$ is still a difficult task, although the prediction accuracy of maximum ${k}_{cat}$ has increased in recent years. The accuracy of DLTKcat is insufficient to quantitatively model bacterial growth and metabolism under different temperature values, while the performance of MPEK on temperature and pH changes is unexamined. Apart from the shortage of temperature and pH-labeled entries in databases, a key bottleneck of predicting temperature and pH dependent enzyme ${k}_{cat}$ lies in the imbalance of datasets particularly at extreme conditions (e.g. pH > 9.0). Similar issues also exist in the prediction of enzyme ${T}_{opt}$, as the dataset used by TOMER and Seq2Topt has fewer than 3000 entries, seldom above 40°C. To tackle this problem, transfer learning might be the potential solution. Enzyme catalytic optimum, turnover number, and protein stability are related to some degree, and thus, an iterative transfer learning strategy could theoretically be implemented to leverage those related datasets. The challenge extends beyond just data volume: available data tends to cluster around well-studied enzymes and their natural variants, leaving sparse coverage for unnatural variants. While data augmentation helps to mitigate the sparsity, it’s still challenging to make predictions for low-identity proteins, proteins with non-canonical amino acids, or novel proteins [116].

The neglect of environmental factors is another shortcoming of existing predictors of enzyme catalytic optimum. Experimental observations suggest that enzyme concentrations in assays, environmental pH, and temperature can affect the measurement of enzyme ${T}_{opt}$ and $p{H}_{opt}$ [117–119], but most datasets used in model development lack such information. Appending relevant metadata via curation from biochemical reaction or protein databases (i.e. BRENDA [120], UniProt [121]) might provide additional information to improve the prediction accuracy of enzyme catalytic optimum.

In addition to transfer learning [112] and statistical data augmentation [103], the integration of biophysics and ML might further advance the prediction accuracy of enzyme catalytic activity. The incorporation of known biophysical constraints and principles into ML models could potentially provide extrapolation beyond the bounds of existing datasets [122, 123]. Physics-based data simulations could augment the dataset with simulation results as a reasonable alternative to experimental data [124, 125]. As such, the combination of statistical and physics-based methods could bridge the gap in currently available data, and guide predictions in sparse regions.

Expectedly, the continuous advancement of ML models of enzyme catalytic activity will address existing limitations discussed in this review and further enhance the prediction accuracy. With accurate identification of enzyme–substrate specificity and estimation of enzyme ${k}_{cat}$ under different pH and temperature, researchers can screen candidate enzymes in a high-throughput way, and adopt predictor-guided generative deep learning to design novel enzymes [126, 127]. Moreover, enzyme ${k}_{cat}$ prediction can be used to fill the gap of missing parameters in enzyme constrained metabolic modeling [68, 92, 128]. Predicted temperature and pH dependent enzyme ${k}_{cat}$ can be used to estimate metabolic pathway capacities under various environmental conditions, enabling environmental condition-sensitive metabolic modeling. Deep learning-driven enzyme-constrained metabolic models can be used to identify rate-limiting steps in cell factories, build precise control models of fermentation kinetics, and investigate inter-species interactions in microbial communities [68, 129, 130]. Therefore, accurate predictors of enzyme catalytic activity will contribute to the construction of computer aided design and optimization pipelines of biocatalysis.

In conclusion, accurate ML models of enzyme catalytic activity hold potential to revolutionize enzyme engineering, metabolic modeling and engineering, and biocatalysis optimization, with advancements in substrate specificity, turnover number, and catalytic optimum prediction paving the way for high-throughput screening and predictor-guided generative design. However, challenges like dataset imbalances and neglected environmental factors persist, underscoring the need for strategies such as transfer learning and continuous data curation to drive further breakthroughs.

**Key Points**

- Recent advancements have been made in ML models for enzyme catalytic activity.
- Attention mechanism, new features, and transfer learning improved prediction accuracy.
- Main limitations exist in data coverage and the neglect of some influencing factors.
- Future breakthroughs will potentially transform enzyme and metabolic engineering.

## Biographical note

Sizhe Qiu is a researcher in the fields of deep learning models of enzymes, metabolic modeling, and multi-omics analysis.

## Data Availability

The code and data are openly available at https://github.com/SizheQiu/MLECA_review_2025.
